# Comparison of ESI and APPI for the Detection of Nitro Explosives Using Differential Mobility Mass Spectrometry

**DOI:** 10.1021/acsomega.6c06952

**Published:** 2026-08-14

**Authors:** Vanessa Lünsmann, René Breuch, Silvia A. M. Herrmann, Kostyantin Konstantynovski

**Affiliations:** † Institute for the Protection of Terrestrial Infrastructures, German Aerospace Center, Rathausallee 12, 53757 Sankt Augustin, Germany; ‡ Hochschule Bonn-Rhein-Sieg, University of Applied Sciences, Institute of Safety and Security Research, von-Liebig-Str. 20, 53359 Rheinbach, Germany

## Abstract

Rapid and reliable detection of explosive residues is critical for security applications (e.g., counterterrorism) and environmental health contexts (e.g., legacy military site contamination). Key target analytes among nitro-organic explosives encompass 2,4,6-trinitrotoluene (TNT), hexogen (RDX), pentaerythritol tetranitrate (PETN) as well as 2,4-dinitrotoluene (DNT, a common military-grade TNT impurity) and 2-amino-4,6-dinitroluene (2A-DNT, a microbial degradation product of TNT). Ion mobility spectrometry is one of the key technologies for fast and reliable detection of explosives. When coupled with mass spectrometry (MS), it enables high sensitivity and precise analyte identification. In this study, a differential mobility analyzer (DMA) coupled to a triple quadrupole MS was utilized to compare two relatively orthogonal ionization techniques—atmospheric pressure photoionization (APPI) and electrospray ionization (ESI)—for explosive trace detection in calibration standards and soil extracts, applied via liquid injection or via thermal desorption from filters. Both ionization techniques showed good linearity and enabled sensitive detection of the tested explosives. While APPI showed increased sensitivity toward dinitrotoluenes (DNT, 2A-DNT), ESI performed significantly better for TNT, RDX, and PETN but failed to detect DNT via thermal desorption. Both ionization techniques were further applied to the analysis of explosives in soil extracts, providing meaningful insights into their applicability for field sample analysis and revealing distinct effects of matrix components on ion source behavior and detection performance. The study underscores the practical relevance of the approach while offering a balanced evaluation of the methodological strengths and limitations.

## Graphical abstract

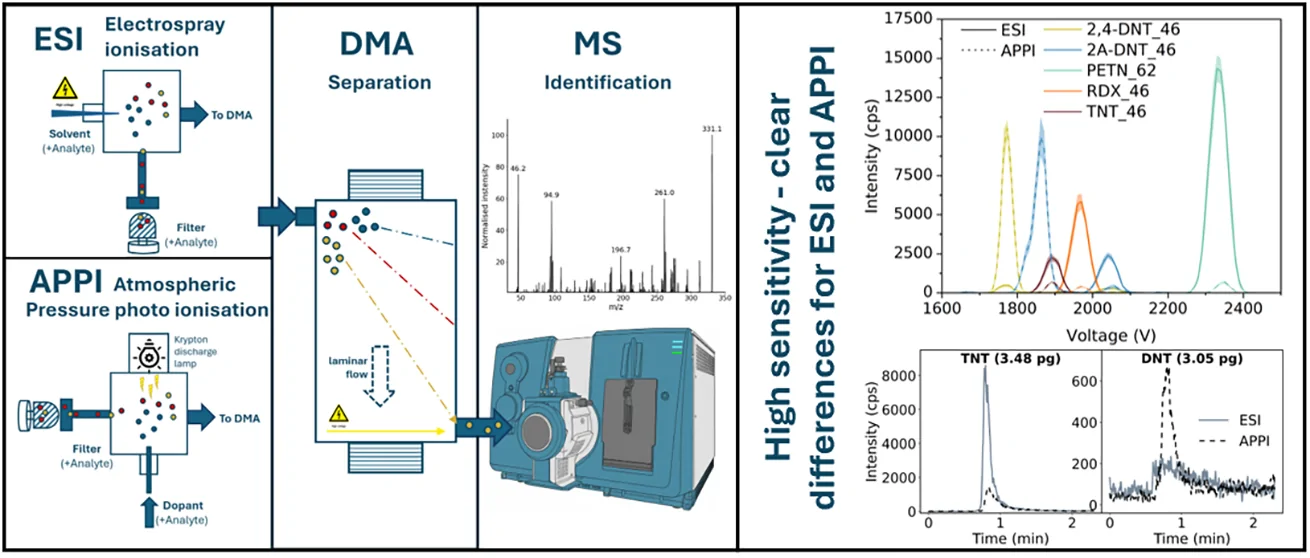

## Introduction

Escalating geopolitical tensions are accelerating the dispersion of military explosives – including TNT – demanding advanced detection technologies to mitigate severe health hazards from explosive contamination in impacted populations and critical infrastructures. Rapid, reliable and sensitive detection of explosive traces is essential for counterterrorism (e.g., airport/border security) and for mitigating health risks from environmental contamination at munition production or military sites.^1–3^ The differential mobility analyzer-mass spectrometry (DMA-MS) platform employed in this study enables subminute detection of explosive residues at trace levels.

The explosives investigated in this study include the nitroaromatic 2,4,6-trinitrotoluene (TNT), its precursor 2,4-dinitrotoluene (DNT), its metabolite 2-amino-4,6-dinitrotoluene (2A-DNT), the nitramine 1,3,5-Trinitro-1,3,5-triazinan (hexogen, RDX), and the nitrate ester pentaerythritol tetranitrate (PETN). Detailed physicochemical properties of all analytes are summarized in Table 1.

**Table 1:**
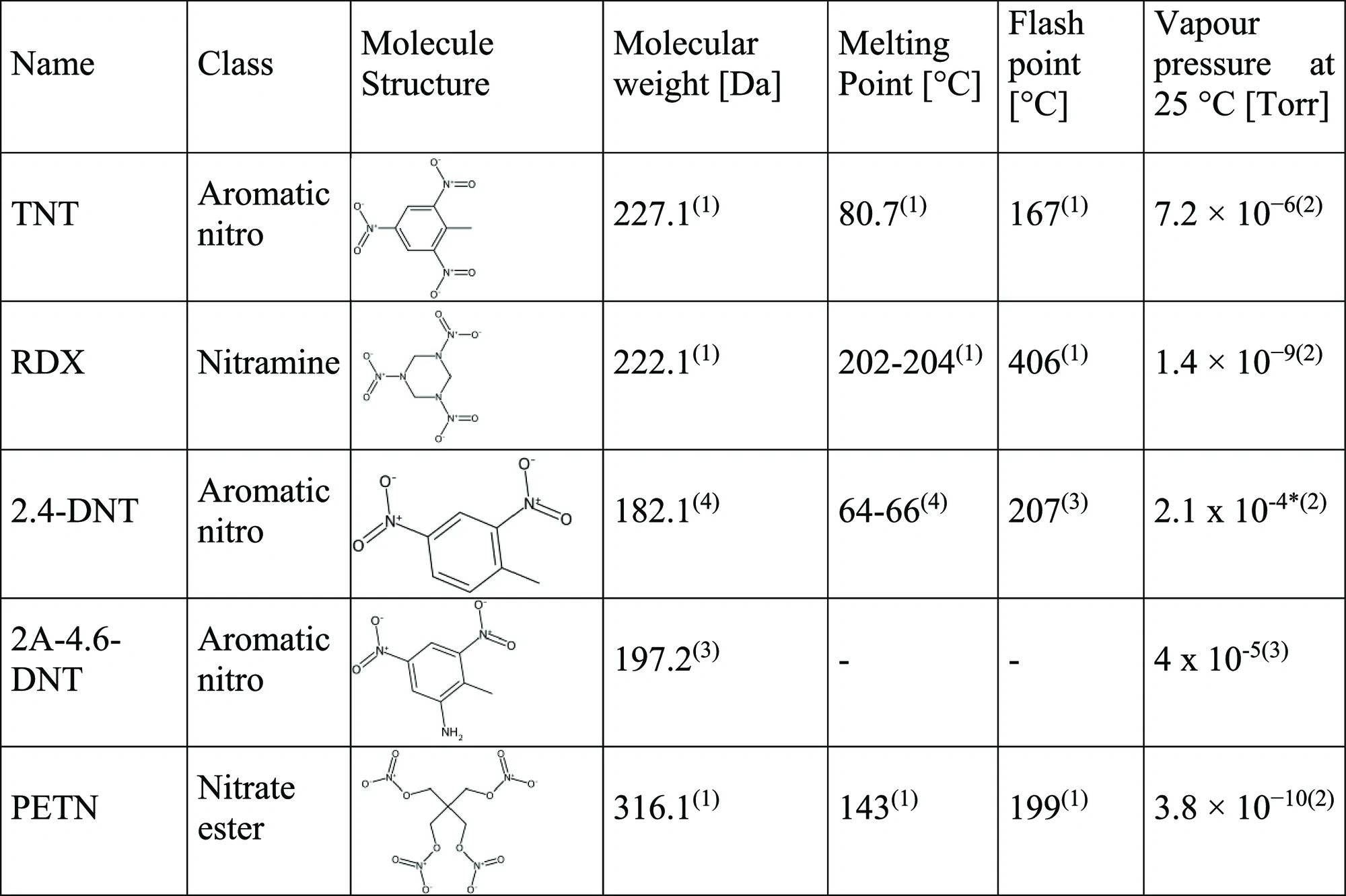
Physicochemical Properties of the Explosives Used in This Study

a 1-2-3-Pubchem, 4-^7^ * = extrapolated.

PETN forms colorless crystals and is one the most powerful and brisant explosives showing satisfactory stability.^4^ Among modern explosives, it has been identified as the least toxic.^5^

RDX is colorless and crystalline, characterized by high stability, insensitivity and significant explosive power due to its high density and detonation velocity.^4^ It is commonly blended with TNT (e.g., Composition B, used as fillings for bombs and mines^4^) or with PETN (e.g., Semtex H) in military formulations.^6,7^ Inhalation or oral exposure to RDX causes nausea and vomiting and the substance is classified as a possible human carcinogen by the US EPA.^8^ Together with TNT, it belongs to the most frequently deployed explosives for military purposes.^7^

TNT, a pale-yellow crystalline solid, is broadly applied for military and industrial purposes attributed to its thermal, chemical, and frictional stability.^4,7^ It is the main explosive charge used in landmines^9^ and the most important explosive for blasting charges in all kinds of weapons.^4^

2,4-Dinitrotoluene (DNT) holds significant relevance as a common impurity in military grade TNT as it is extensively employed in the production of explosives and propellants, serving as a gelatinizing, plasticizing, and waterproofing agent.^10,11^ Due to its higher vapor pressure and chemical stability, DNT often co-occurs with TNT and is considered a marker substance for TNT-based explosives.^12^ Another key metabolite of TNT, 2-amino-4, 6-dinitrotoluene (2A-DNT) is a primary reduction product and commonly detected in TNT-contaminated soils.^13^ TNT and its derivatives DNT and 2A-DNT are classified as possible human carcinogens.^14,10^

Soil contamination by explosives is a global concern, especially in Africa and Asia, due to sources like manufacturing effluents, unexploded ordnances (UXOs), mining, and military testing.^15^ The persistence and mobility of explosives in soils depend on their physicochemical properties as well as environmental factors such as temperature and soil composition. While TNT is rapidly transformed into soil-accumulating amino-DNTs, RDX persists strongly in soils by adsorbing to particles. Both explosives cause long-term contamination with adverse effects on soil quality, impairing plant growth and root development.^12,16^ Given these risks, soil pollution by explosives represents a serious environmental concern with potential implications for ecosystem integrity and human health.^16^ Consequently, the accurate detection of explosive residues in soil is crucial for effective environmental monitoring and successful implementation of site remediation strategies.^17^

Among the various technologies for explosive detection, ion mobility spectrometry (IMS) has been employed for decades – particularly in aviation security – and continues to gain increasing importance.^20^ It is a powerful technique for chemical trace detection across diverse fields, including chemical weapon monitoring, air quality analysis as well as biological and medical diagnostics.^21^ The majority of commercially available IMS instruments is however applied for the detection of explosives at security checkpoints worldwide.^22,23^ While numerous types of IMS instruments exist (comprehensively summarized in^21,24^ ), the fundamental principle always involves the ionization of vapors and the subsequent separation of resulting ions based on their size-to-charge ratio in an electric field under ambient or reduced pressure conditions.^21,25^ Whereas conventional IMS instruments typically employ a Faraday plate for detection providing only limited information about the ions,^22^ coupling IMS with mass spectrometry (MS) adds a key separation dimension based on *m*/*z*, enhancing sensitivity and enabling unambiguous ion identification.^26^ Further comprehensive general information on ion mobility spectrometry can be found in dedicated publications e.g., by^27^ and^28^.

One specialized IMS variant is the differential mobility analyzer (DMA), its development and theoretical foundations are thoroughly reviewed in^29^. Similar to other IMS techniques, DMA can also be combined with MS, to further increase selectivity and sensitivity.^30,31^ In the DMA-MS system employed in this study, ions are migrating between two parallel electrodes, facilitated by a laminar gas flow, eventually passing through the exit slit and entering the mass spectrometer. By modulation of the electric field through the application of a specific voltage, the DMA acts as an ion filter, selectively permitting only ions of interest to reach the detector.^21^ Additionally, by applying a voltage gradient across a range of values, analyte ions can be separated from “matrix ions” based on their distinct mobilities in less than a minute. The combination of a planar DMA and a triple quadrupole MS (similar system) has already been successfully applied for the trace detection of explosive vapors.^32^

Ionization is a critical step in both IMS (DMA) and MS, and these techniques can be coupled with various ionization principles. For IMS, any ionization method compatible with ambient pressure is applicable^33^ with ^63^Ni being the most widely used ion source.^23^ However, regulatory and operational constraints associated with radioactive materials have driven the development of nonradioactive alternatives, particularly vacuum ultraviolet photoionization.^23^

Atmospheric pressure photoionization (APPI) typically employs a krypton discharge lamp emitting photons at 10.0 and 10.6 eV. While theoretically ionization only requires analytes with ionization energies (IE) below the energy of the lamp emission, direct photoionization is often inefficient.^34^ To enhance ionization efficiency, a dopant solvent – such as acetone, toluene or isoprene – with an IE below the energy of the emitted photons is introduced. The dopant absorbs the photon energy and further transfers the resulting charge to the analyte molecules.^35^ In case of negative ionization – as it was applied in this study – thermal electrons released from the excited dopant molecules or metal surfaces (electrode) in the ion source are captured by molecules with high electron affinity. At atmospheric pressure, however, these electrons are often captured by oxygen, forming superoxide ions (O_2_^–^) which can subsequently ionize the analytes through various pathways such as charge transfer (yielding M^–•^) or deprotonation (yielding [M-H]^−^). When halogenated solvents (e.g., dichloromethane) are used as dopants or additives, anion attachment results in halide adducts [M+X]^−^.^36^ Further information on APPI-IMS as well as details of the ionization mechanisms can be found in.^37^

In contrast to APPI, which is a gas phase ionization technique, electrospray ionization (ESI) relies on liquid phase ionization,^38^ is widely used in MS and also adapted for IMS.^39^ In principle, an analyte solution is pumped through a capillary with a high voltage (3–5 kV) applied. This strong electric field, combined with a supporting gas flow, transforms the liquid into a fine aerosol spray of charged droplets. As the droplets move toward the DMA entrance, solvent evaporation due to temperatures above 100 °C lets them shrink, ultimately resulting in gas-phase ions (“ion evaporation”).^40^ There are three different models explaining the process of ion generation which are beyond the scope of this study but can be found in Liao et al.^41^ Typically, ESI produces [M-H]^−^ or [M + H]^+^ ions, though contaminants such as sodium or ammonium ions may cause adduct formation. By directed addition of modifiers, the ionization process can be modulated to increase the intensity of the desired adduct ions. A subtype of ESI is secondary ESI (termed SESI), where the analyte molecules are not part of the ESI spray but are delivered separately as vapor (e.g., via thermo desorption from filters) or aerosol. The ionization mechanism of SESI is not fully understood, with most studies focusing on positive ionization. However, two ionization mechanisms come into consideration: (1) gas-phase-ion molecule reactions, resembling the ionization mechanism of APCI; (2) vapor-droplet interactions, involving the dissolution of analyte molecules into the charged ESI droplets and their subsequent ionization through an ESI process. A series of studies revealed that gas-phase-ion molecule interactions dominate the ionization process.^41^

The ionization techniques described above have been used in combination with differential mobility mass spectrometry, e.g., for ESI,^42^ ESI and APCI,^43^ APPI^44^ and ESI, APPI, APCI,^45^ but a direct comparison of their performance for explosive detection when coupled to a DMA-MS has not been described in literature, yet.

### Aim

Subsequently, this study evaluates the sensitivity and performance of ESI and APPI when coupled to a DMA-MS for the detection of explosives. By comparing typical mobility spectra and calibration curves, we assess the suitability of each ionization technique. The ionization techniques evaluated were subsequently employed in the analysis of explosives within soil extracts, marking the system’s first practical application for detecting explosive residues in contaminated soils.

## Materials and Methods

### Chemicals

LC-MS grade water, acetone, acetonitrile (ACN), dichloromethane and methanol (MeOH) were purchased from Th. Geyer (Germany).

Explosive standard solutions (TNT, PETN, RDX, each 100 μg/mL in methanol or acetonitrile/methanol) were obtained from amchro GmbH (Hattersheim a.M., Germany). Standards of 2,4-DNT and 2-amino-4,6-DNT were purchased from HPC Standards GmbH (Cunnersdorf, Germany). All standards were further diluted in acetonitrile/methanol (50/50 v/v) to obtain the concentrations listed in Table 3.

Vanillin (99% purity) was obtained from ThermoFisher Scientific (Schwerte, Germany). 10 mg were dissolved in methanol and further diluted in methanol/water (50/50 v/v). Vanillin was added as internal “mobility standard” to the ESI solvent or the APPI dopant to a final concentration of 1 μg/L.

### Soil Samples

Standardized soil (2.4N, soil type clay loam) was obtained from LUFA Speyer (Speyer, Germany). Four portions of 2 g soil were weighed separately into 15 mL PP tubes (Sarstedt, Nürmbrecht, Germany). Three replicates were fortified with 20 μL of ACN/MeOH (50/50 v/v) containing TNT (0.12 mg/L), RDX (0.40 mg/L) and DNT (0.20 mg/L) in order to obtain soil samples fortified with TNT, RDX and DNT at 0.0012, 0.0020 and 0.0040 mg/kg, respectively. One blank sample was spiked with 20 μL of ACN. All samples were briefly vortexed and placed into an overhead shaker at 80 rpm for 10 min. For extraction, 4 mL of ACN were added, samples were briefly vortexed and placed into the overhead shaker at 80 rpm for 10 min, followed by two intervals of 10 min in an ultrasonic bath (Sonorex Digitec, Bandelin, Berlin, Germany). Afterward, the samples were centrifuged for 5 min at 5000 rpm (Universal 320R, Hettich GmbH, Tuttlingen, Germany) and extracts were filtered (Chromafil Xtra PA-20/25, Machery-Nagel GmbH & Co. KG, Düren, Germany) into 4 mL glass vials (ND13, LABSOLUTE, Th. Geyer, Renningen, Germany). Extracts were kept frozen (≤ −18 °C) until analysis.

### DMA-MS

A TQ5500+ triple quadrupole mass spectrometer (SCIEX, MA) in negative ion mode was used for all experiments (backing pump Agilent MS40+ (Agilent Technologies Inc. CA)). The system was coupled to the planar P5-f DMA (MION Technologies, former SEADM) instead of its usual ion source. A very similar DMA is described in detail in^43^, the coupling between DMA and the MS is described in^44^. The DMA temperature is not controlled, it was usually operated at around 40 °C. The sample flow was fixed at 0.2 L/min (flow from thermo desorber to the ion source), the output flow was 1.3 L/min, the DMA blower – causing the laminar flow in the DMA – was set to 13 000 rpm. The system was provided with nitrogen (99.5% purity) from a nitrogen generator (NGM 11, cmc Instruments GmbH, Eschborn, Germany).

Attached to the DMA and operating as an ion source for the DMA-MS were either an APPI (described in^44^ ) or an ESI ion source (low-flow-desolvating ESI, described in^43^). An overview of the different DMA-MS sections is presented in Figure 1.

**Figure 1:**
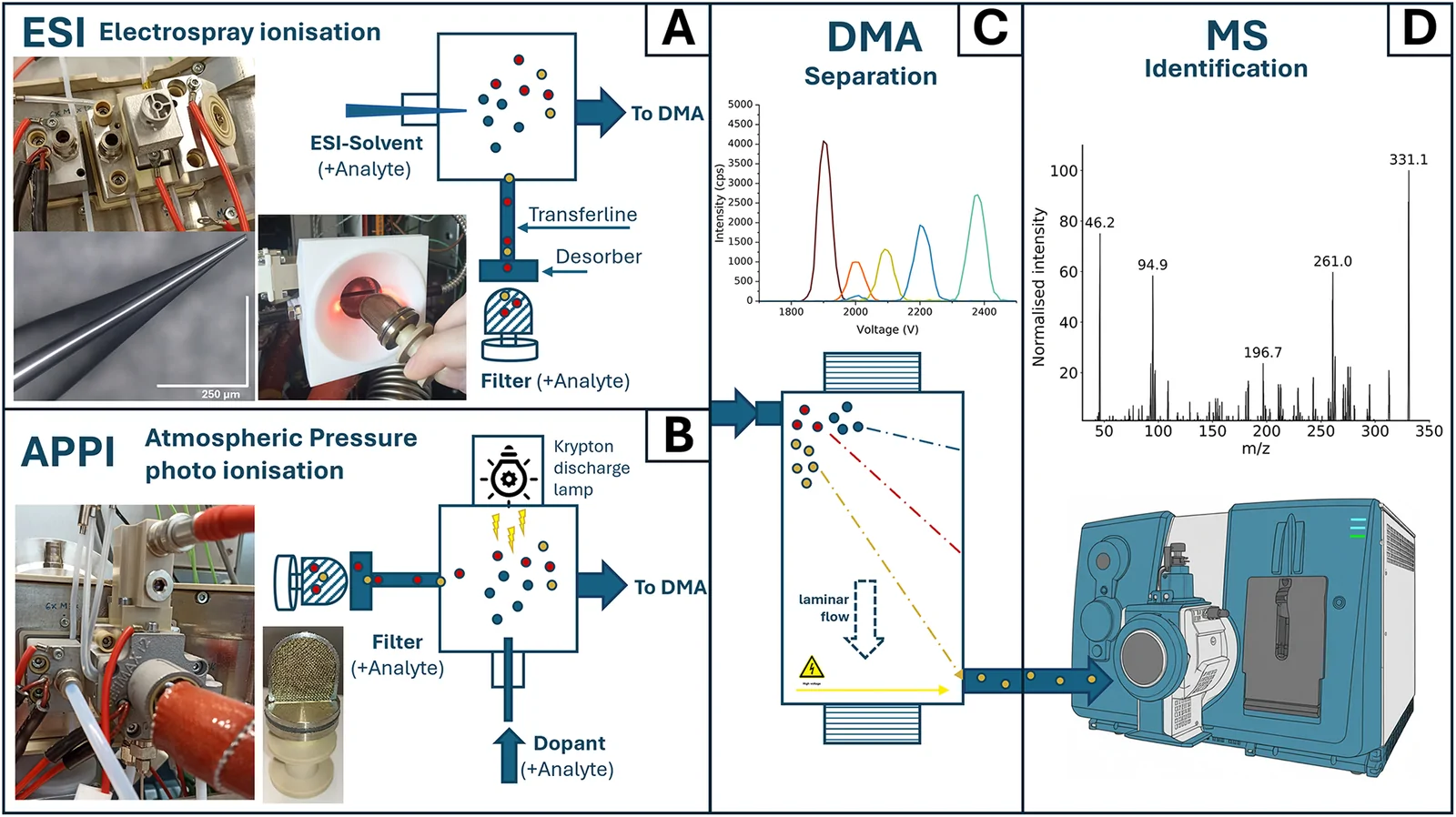
(A) Scheme and photograph of the ESI-source, a microscopic photograph of an ESI-emitter-tip and the insertion of a filter into the desorber. (B) Scheme and photograph of the APPI-source and a photograph of the filters used for the measurements. (C) Scheme of the DMA and an exemplary mobility spectrum. (D) Scheme of the MS.

#### ESI-Source

The electrospray ionization source (MION Technologies, Valladolid, Spain) described in^43^ was operated at 160 °C, spraying a solvent consisting of methanol/water (9/1 v/v) containing 0.04% v/v HCl and 1 μg/L vanillin (ESI solvent). ESI emitter tips were obtained from MS Wil (Aarle-Rixtel, Netherlands; fused silica 50 μm ID × 65 cm; 365 μm OD Orifice, ID 15 μm; CoAnn Technologies LLC). The solvent was delivered by generating pressure on the liquid reservoir (ND18, 10 mL glass vials, LABSOLUTE, Th. Geyer, Renningen, Germany). The average flow rate was determined by weighing the solvent vial before and after constant operation for 24 h as 1.26 μL/min.

#### APPI-Source

The atmospheric pressure photoionization source (MION Technologies, Valladolid, Spain) described in^44^ was operated at 0.9 L/min nebulizer flow, with a temperature gradient inside the ion source of EL3:160 °C and EL2:150 °C. The dopant was delivered by the syringe pump of the MS, using a 1 mL Hamilton Syringe (Hamilton, NV) at a flow rate of 6 μL/min. A PEEK capillary (1/16”, 0.1 mm ID; VICI AG International, Schenkon, Switzerland) was used to deliver the dopant (5% v/v dichloromethane in acetone containing 1 μg/L of vanillin) from the syringe to the ion source. The actual photoionization was achieved by a 10.6 eV krypton lamp (Heraeus, 80017644).

#### DMA

The DMA Can Be Operated in Different Modes: Voltage scan: the voltage of the DMA is ramped with a fixed step (e.g., from 1500 to 2700 V, step of 10 V). This mode was used for the analysis of liquid samples. Within each step, all respective mass transitions (Table 2) are analyzed for the dwell time given in the MS method.

**Table 2:** Mass Transitions and Instrument Parameters for the Analyzed Substances^a^

Analyte	Q1 (Da)	Q3 (Da)	ID	DP (V)	CE (V)	CXP (V)
2.4.6-TNT [M-H]^−^	226	46*	TNT_46	0	–70	–8
	226	166	TNT_166	0	–24	–8
2.4-DNT [M-H]^−^	181	46*	2,4-DNT_46	0	–53	–20
	181	135	2,4-DNT_135	0	–38	–20
2A-DNT [M-H]^−^	196	46*	2-ADNT_46	0	–52	–21
	196	136	2-ADNT_136	0	–22	–21
PETN [M+Cl]^−^	351	46	PETN_46	0	–40	–7
	351	62*	PETN_62	0	–20	–7
RDX [M+Cl]^−^	257	46*	RDX_46	0	–30	–10
	257	82	RDX_82	0	–10	–10
Vanillin [M-H]^−^	151	92	vanillin_92	–50	–29	–10
	151	136*	vanillin_136	–50	–17	–10

a Quantifier marked *, entrance potential (EP) was 10 V for all analytes.

Voltage sweep: several different, fixed voltages are successively applied and the DMA is capable of changing between these voltages within milliseconds. When using an MRM-method with the MS, for each analyte, the specific optimum voltage is given and the DMA applies the respective voltages synchronized with the dwell time of each MRM.

Vanillin was used as internal standard in order to determine the optimal DMA voltages for the analyzed explosives. The exact voltages can be slightly different from day to day, the ratio of the voltages from the internal standard and the analytes is stable.^32^

#### Mass Spectrometry

The triple quadrupole was operated in multiple reaction monitoring (MRM) mode, using the parameters given in Table 2 (previously optimized): Analyst Version 1.7.2 was used for data acquisition and peak integration.

### Measurement of Liquid Samples

In principle, liquid sample measurements are used for method development such as determination of the optimum passage voltage and mass transitions of the analytes. For the preparation of the samples, explosives were spiked in the corresponding solvents used for ESI or APPI at the concentrations listed in Table 3. The total volume of all analytes added to the ion source solvents was kept below 1% of the final solution. This ensured that the solvent composition necessary for each ionization type was maintained. For each explosive, the most intense mass transition (quantifier, determined from measurements of both transitions and marked with * in Table 2) was monitored with a dwell time of 200 ms while the DMA voltage was ramped from 1500 to 2700 V in order to obtain a mobility spectrum as depicted in Figure 2 (voltage scan). Additionally, blank measurements (no explosive in the solvent) were performed.

**Table 3:** Concentrations of Explosives for Liquid Measurement

Analyte	APPI (μg/L)	ESI (μg/L)
TNT	1.99	1.99
DNT	5.09	106.7^a^
RDX	0.99	0.99
PETN	0.99	0.99
2A-DNT	2.00	51.9^a^

a Elevated concentration levels due to reduced sensitivity.

### Measurement of Filter Samples

For determination of TNT, DNT and RDX from filter samples via the thermo desorber, calibration standards were prepared in methanol/acetonitrile (50/50 v/v). All three analytes were combined in one calibration solution. For each measurement, 5 μL of the respective calibration standard (or soil extract) were pipetted onto a filter (stainless-steel, glass fiber coated with Tenax-GR; MION Technologies, Valladolid, Spain; compare Figure 1). The measurement of the DMA-MS was started (voltage sweep; fixed voltage for each mass transition; method shown in Table 5), and after the first 0.6 min, the filter was placed into the thermo desorber (200 °C) for analysis. The filter was left in the desorber for 1.7 min. During this time, the filter is flushed by a constant flow of nitrogen, transporting the desorbed sample vapors through the transferline to the respective ion source. All samples were analyzed in eight replicates. Several times on each measurement day, the optimal voltage of vanillin was checked and the voltages for the explosives were adapted, accordingly. The analyzed concentrations are listed in Table 4.

**Table 4:** Calibration Standards for Filter Measurements

		Concentration (μg/L)			Amount Deposited onto Filter (pg)		
Standard	Used for	TNT	DNT	RDX	TNT	DNT	RDX
Cal 1	ESI	0.50	0.76	1.49	0.099	0.152	0.298
Cal 2	ESI	1.24	1.90	3.72	0.248	0.381	0.744
Cal 3	ESI/APPI	2.48	3.81	7.44	0.497	0.762	1.488
Cal 4	ESI/APPI	4.97	7.62	14.88	0.993	1.524	2.976
Cal 5	ESI/APPI	9.93	15.24	29.76	1.986	3.048	5.952
Cal 6	ESI/APPI	17.38	26.67	52.08	3.476	5.334	10.42
Cal 7	ESI/APPI	24.83	38.10	74.40	4.965	7.620	14.88
Cal 8	ESI/APPI	37.24	57.15	111.60	7.448	11.43	22.32
Cal 9	ESI/APPI	49.65	76.20	148.80	9.930	15.24	29.76

### Data Treatment

Peak heights and areas were determined from the unsmoothed data using Analyst 1.7.2. For peak integration, a consistent approach was adopted, taking into account the desorption behavior of the analytes, background signals and the time when the filter was inserted into the desorber. From the start of the desorption, peaks for TNT and DNT were integrated for 0.6 min, peaks of RDX were integrated until the end of the measurement (about 1.7 min). The result tables were exported using Excel (Office Professional Plus 2024), OriginPro 2025 (Version 10.2.0.196) was used for further processing (e.g., linear regression). The wiff-files obtained from DMA-MS-measurements were converted to mzml-files using MSConvert (version 3.0.25017 abe9cec). Mzml-files were imported to OriginPro 2025 (Version 10.2.0.196) for plotting and statistical analysis. Limits of detection (LOD) were calculated from eight replicate blank measurements for both ion sources using Excel according to the formulas given in the Results section. For graphic representation of signals from filter measurements, data were Gaussian smoothed with the Savitzky-Golay method (window length = 5, sigma = 2) of python’s scipy package.

**Table 5:** MS-Method for Analysis of Filter Samples (Total Duration: 2.3 min)^a^

Analyte	Q1 (Da)	Q3 (Da)	Dwell Time (ms)	ID	DP (V)	EP (V)	CE (V)	CXP (V)	DMA Voltage Ratio (Analyte/Vanillin)
2.4.6-TNT [M-H]-	226	46	100	TNT_46	0	–10	–70	–8	0.964
RDX [M+Cl]^−^	257	46	100	RDX_46	0	–10	–30	–10	1.011
2.4-DNT [M-H]^−^	181	46	100	2,4-DNT_46	0	–10	–53	–20	0.910
vanillin [M-H]^−^	151	136	25	vanillin_136	–50	–10	–17	–10	1

a DMA voltages were adapted on each day according to the optimum voltage for vanillin.

## Results

### Solvent Measurements

The comparison of the two ion sources was initiated by introducing the analyte mixture into the DMA-MS via liquid-phase application. Figure 2 shows voltage scans from replicate solvent measurements (*n* = 8: mean (line) and standard deviation (SD; depicted as area)) for both ion sources, acquired in MRM-mode (compare Table 2). In principle, the DMA functions comparably to a chromatography: Each explosive exhibits its optimum DMA passage voltage (peak maximum) and in the range of approximately ± 50 V around the optimum voltage, fractions of the explosive molecules are still able to reach the MS, resulting in more or less “classical” peak shapes as known from chromatography (mobility spectra). Signals of the APPI measurements were normalized according to the different flow rates (APPI: 6 μL/min; ESI: 1.26 μL/min) and different concentrations of the analytes between the ion sources (compare Table 3).

**Figure 2:**
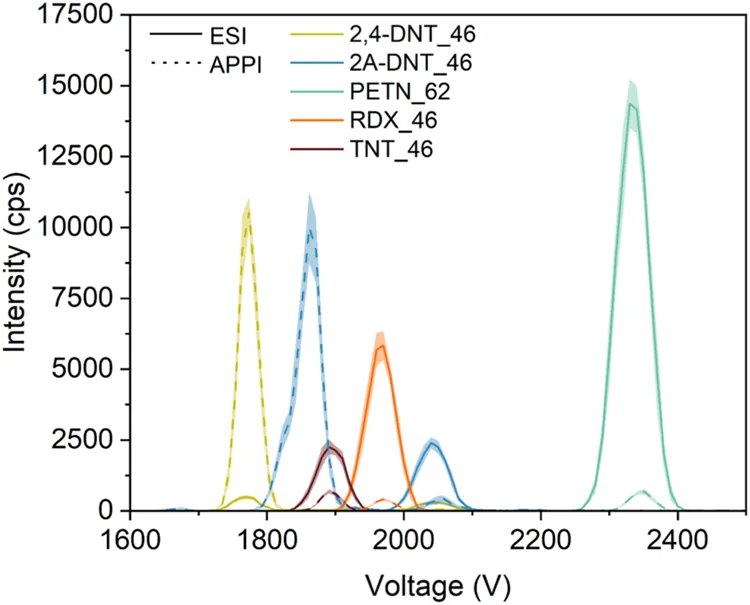
Mean intensities and standard deviation for a voltage scan of explosives dissolved in liquid suitable for each ionization method (*n* = 8 for each ionization method).

From the figure, ion source-dependent distinctions are clearly visible: While ESI performs much better for TNT, RDX and PETN (factors 3, 14 and 21, respectively (considering peak heights)), APPI shows increased sensitivity toward 2,4-DNT and 2A-DNT (factors 22 and 4, respectively (considering peak heights)). For both ion sources, TNT, RDX and PETN exhibit one clear peak, while the behavior of DNT and 2A-DNT is more complex: With the ESI source, DNT appears as two separated peaks (maxima at 1770 and 2040 V), while with the APPI source, DNT shows only one clear peak at 1770 V. For 2A-DNT, the optimum passage voltage differs strongly between the two ion sources: with the ESI source, the maximum is at 2040 V, while it shows two distinct peaks when analyzed with the APPI source: the main peak is at 1860 V, and a secondary peak is visible at 2040 V.

### Filter Measurements

For the detailed evaluation of the instrument’s performance regarding the detection of soil-related explosives, we focused on TNT and RDX, as these are commonly detected in contaminated soils.^16^ While 2A-DNT would be a very important candidate for determination of soil contamination with TNT, its detection from filters using the thermo desorber has not been established, yet and is part of ongoing research. However, DNT was included as an important metabolite of TNT. Figure 3 shows representative desorption curves from filter measurements for TNT, RDX and DNT. RDX exhibited broader desorption profiles compared to TNT and DNT. The higher sensitivity of ESI for RDX and TNT is reflected in greater signal intensities for equivalent explosive deposits. While DNT was detectable by liquid measurements with both ionization sources — APPI demonstrating superior sensitivity for DNT relative to TNT (Figure 2) — APPI’s sensitivity for DNT significantly diminished during filter analysis. Notably, DNT analysis via the ESI source resulted in an elevated baseline with no detectable peak upon filter introduction, regardless of the concentration level used (Figure S1). Due to the decreased detectability of DNT, the prevalence of other precursor ions (M^–•^ = 182 *m*/*z* or [M+Cl]^−^ = 217 *m*/*z*) was checked but could not be confirmed.

**Figure 3:**
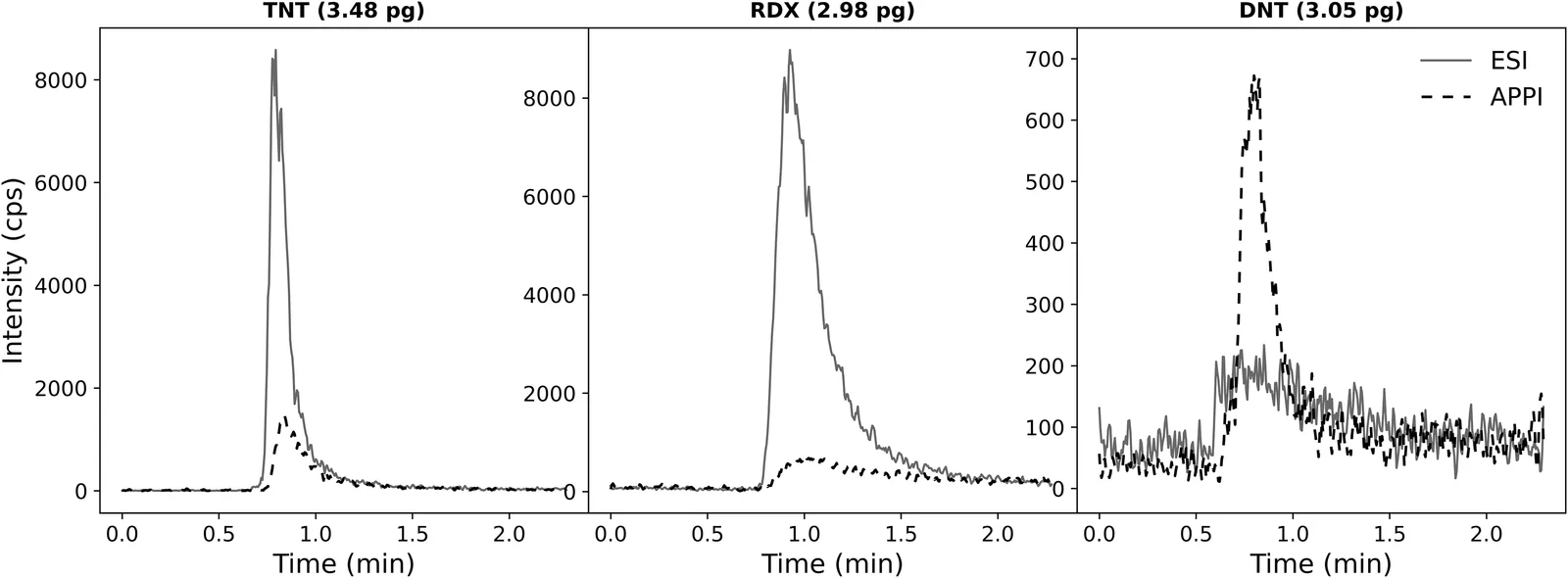
Desorption curve for filter measurements of standards with a medium concentration (about 3 pg of each explosive). Signals were Gaussian smoothed using the Savitzky-Golay method (sigma = 2, window length = 5).

### Linearity and LOD

To further investigate the performance and linearity of the detection method, calibrations curves were recorded for the three explosives TNT, RDX and DNT. Figure 4A shows an exemplary regression curve for TNT analyzed by ESI (eight replicates per concentration level) alongside comparative ESI and APPI calibrations on full-logarithmic scale (Figure 4B). The calibrations exhibited standard deviations proportional to the mean, exhibiting no clear trend (Table S1). Therefore, a weighted linear regression (1/σ_i_^2^) was performed using OriginPro 2025 (Version 10.2.0.196) with the weight inversely proportional to the square of the errors, giving greater influence to more precise measurements, with all calibrations demonstrating good linearity (*R* ^2^ > 0.98). Table 6 summarizes regression parameters for all analyte-ion source pairs. ESI covered a two-order-of-magnitude concentration range, while APPI spanned one order. The highest calibration points were consistent across methods: 9.93 pg (TNT), 15.2 pg (DNT), and 29.8 pg (RDX).

**Figure 4:**
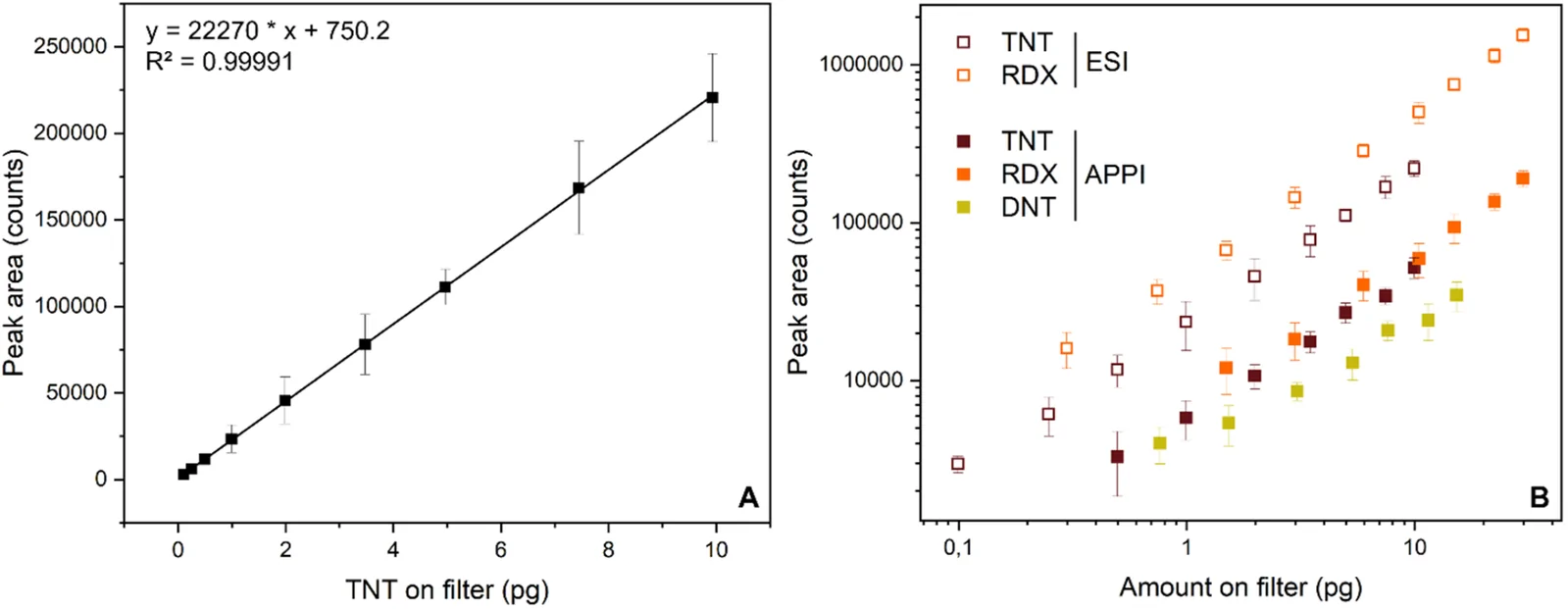
(A) Exemplary regression curve for TNT analyzed by filter measurements using ESI (peak area). (B) Comparative calibration standards (mean and standard deviation), depicted on a full-logarithmic scale for TNT, RDX and DNT.

**Table 6:** Parameters of the Linear Regression for All Analytes and Ion Sources

Source	Analyte	m	S_m_	b	S_b_	*R* ^2^
APPI	RDX	6101	177.3	1942	1169	0.99579
	TNT	4829	182.2	1008	477.1	0.99293
	DNT	2158	119.6	2206	397.2	0.98488
ESI	RDX	49514	885.2	–80.79	1460	0.99777
	TNT	22270	81.99	750.2	23.85	0.99991
	DNT^a^	–	–	–	–	–

a Detection was not possible.

To finally describe the limitations of both methods for the detection of the explosives, the limits of detection (LOD) were calculated according to three different common approaches recommended by the ICH guideline,^46^ relying on peak areas.

Based on signal-to-noise (simplest approach)

$$X_{LOD\left(a\right)}:S/N>3$$

X_LOD(a)_: Limit of detection. S_blanks_: Standard deviation of the blank signals.

Based on standard deviation of the blank

$$X_{\mathrm{LOD}\left(b\right)}=3^{*}S_{\mathrm{blanks}}$$

X_LOD(b)_: Limit of detection. S_blanks_: Standard deviation of the concentrations determined in blank samples.

Based on standard deviation of the blanks and the slope of the calibration curve

$$X_{\mathrm{LOD}\left(c\right)}=3.3^{*}\frac{S_{\mathrm{blanks}}}{m}$$

X_LOD(c)_: Limit of detection. S_blanks_: Standard deviation of the blank signals. *m*: Slope of the calibration curve.

Table 7 shows the calculated LODs for the three analytes for both ionization procedures. Except for DNT, which could not be detected on the filters using the ESI source, the LODs are lower for ESI than for APPI. Especially the sensitivity for TNT is increased by a factor of 5–9 (depending on the LOD formula applied) when using the ESI instead of the APPI-source.

**Table 7:** Estimated LODs (Given in pg) for the Different Analytes Using Three Different Approaches for LOD Determination

LOD Method	LOD (pg)					
	TNT		RDX		DNT	
	ESI	APPI	ESI	APPI	ESI^a^	APPI
(a)	0.006	0.046	0.017	0.358		0.103
(b)	0.023	0.208	0.178	0.335		0.498
(c)	0.052	0.259	0.203	0.401		1.351

a Detection was not possible.

### Soil Extracts

To assess the applicability of both ionization sources for the detection of explosives in contaminated soils, standardized soil samples were fortified with explosives, extracted and analyzed using the filter assisted thermal desorption of the DMA-MS with ESI and APPI ion sources. Figure 5 presents the recoveries (analyzed concentration/nominal concentration × 100%) for three fortified soil samples, each analyzed in eight replicates. ESI consistently yielded recoveries exceeding 100% (mean ± SD: TNT 128 ± 9.8%, RDX 106 ± 9.9%), indicating overestimation of analyte concentrations while showing acceptable standard deviations below 10%. In contrast, APPI produced recoveries below 100% (mean ± SD: TNT 81.3 ± 11%, RDX 85.5 ± 15%, DNT 72.5 ± 23%), suggesting underestimation of sample concentrations and showing higher standard deviations compared to ESI. These results highlight ion source-dependent matrix effects in analysis of soil extracts.

**Figure 5:**
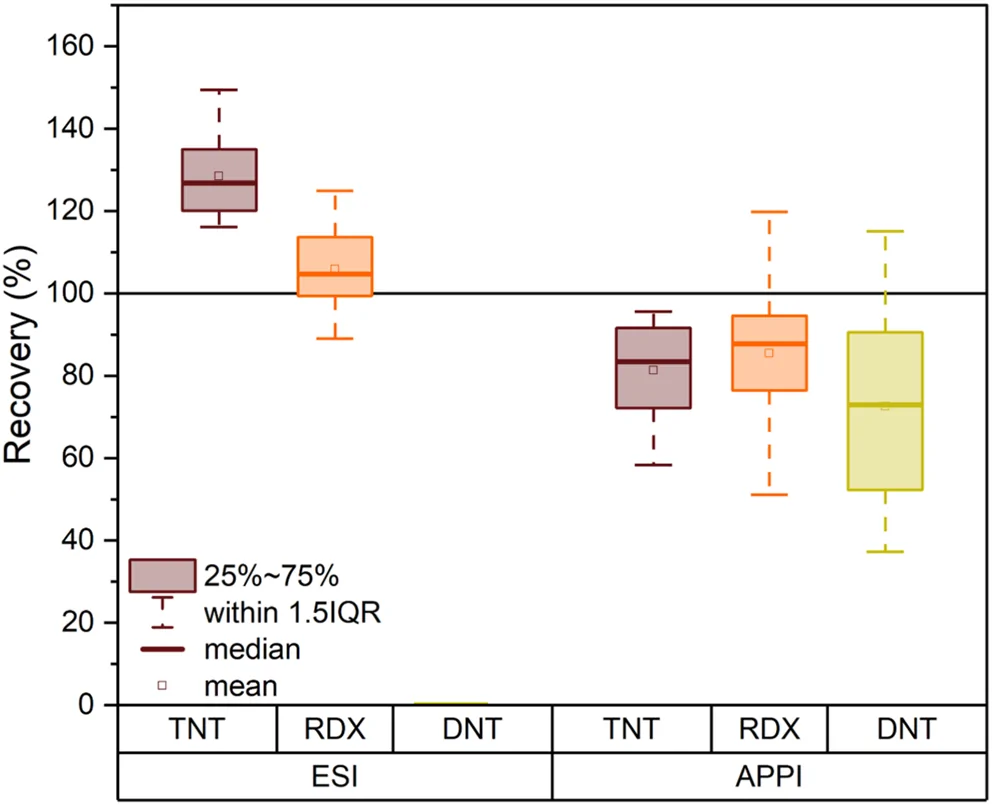
Boxplots of recoveries of the analytes in the analyzed soil extracts (*n* = 24 for each box plot).

## Discussion

The experimental results revealed clear differences between the two ion sources. The effectiveness of each ion source varied depending on the analyte as well as the nature of the measurement task. For a comprehensive evaluation of ESI and APPI, the comparison was carried out across multiple criteria including the mode of measurement and key analytical parameters such as linearity and LOD. Finally, both ion sources were compared regarding their potential for the analysis of soil extracts.

### Solvent Measurements

Liquid-phase mobility spectra revealed distinct source-dependent patterns for the five analytes. APPI demonstrated superior sensitivity for DNT and 2A-DNT, whereas ESI exhibited enhanced response for TNT, RDX, and PETN – consistent with findings that ESI favors nitramines and nitrate esters over nitroaromatics due to gas-phase acidity constraints.^47^

The observed mobility order (DNT < TNT < RDX < PETN) aligns with physicochemical properties of the analyzed ions. The mobility correlates with ion size, charge, and shape.^48^ Notably, RDX was analyzed as chloride adduct ([RDX+Cl]^−^), elevating its mass relative to [TNT-H]^−^ despite RDX’s lower molecular weight. The ordering of the five explosives is further corroborated by their reduced mobility *K*_0_, determined in^49^.

Optimum passage voltages as well as the occurrence of multiple peaks for one analyte were also source-specific: At 2040 V, ion clusters for DNT (ESI secondary peak) and 2A-DNT (sole ESI peak, APPI secondary peak) were observed. For 2A-DNT, the optimum passage voltage with APPI was at 1860 V. These effects are probably attributable to divergent gas-phase chemistries induced by source-specific solvents, promoting distinct ion clustering/solvation states, altering the mobilities of the analytes.^50^ Subsequent desolvation or declustering of DNT and 2A-DNT in the MS interface still enabled accurate detection. Possible clusters or adducts that could have occurred are among others [M+Ac]^−^, [M+NO_3_]^−^, [M+NO_3_]^−^ or [M+Cl]^−^. As the formation of chloride adducts is supported by both ion source solvents (both containing chloride), other adducts might still occur as they are present as impurities in glassware, solvents and ambient air^51^ or – in case of NO_3_ and NO_2_ – might even originate from the partial decomposition of the tested explosives, especially RDX.^52^ The possible formation of chloride adducts for DNT is confirmed by^44^, who also found the [M+Cl]^−^-ion for 2,6-DNT. Despite all possible adduct ions, it would be surprising to find exactly the same optimum passage voltage for 2A-DNT and DNT (or their respective adducts), as both molecules differ from each other. In this context, it has to be noted that the voltage gradient was performed in steps of 10 V, so mobility differences smaller than 10 V cannot be determined.

### Filter Measurements

Desorption profiles for TNT and RDX aligned with their respective vapor pressures (3.0 × 10^–6^ Torr and 1.4 × 10^–9^ Torr, respectively; Harper et al., 2005): TNT exhibited a narrow, rapid desorption peak, whereas RDX – characterized by lower volatility – displayed delayed onset (by several seconds) and prolonged desorption kinetics. Elevated desorption temperatures (>200 °C) could enhance RDX signal intensity, however, the design of the desorber does not allow such optimization.

Filter-based measurements revealed distinct sensitivities across ion sources and showed a deviating DNT response relative to liquid-phase analyses: despite its higher vapor pressure (2.1 × 10^–4^ Torr) compared to RDX and TNT, DNT demonstrated lower sensitivity with APPI than anticipated, suggesting additional factors influencing analyte desorption and transfer-line transmission efficiency. Furthermore, ESI completely failed to detect DNT from filter samples, contrasting with successful liquid-phase analysis.

### Linearity and LOD

Calibration curves for both ESI and APPI utilized identical upper limits (TNT: 9.93 pg; DNT: 15.2 pg; RDX: 29.8 pg), all well below detector saturation threshold or reactant ion limitation. This is evidenced by the linear response across all combinations of analytes and ion sources. Full-logarithmic calibration plots revealed uniformly distributed relative standard deviations, which are marginally elevated for low-level APPI standards – attributable to signal intensities approaching the noise floor, where stochastic fluctuations exert greater relative influence. To address concentration-dependent variance (heteroscedasticity), all calibration curves were fitted using 1/σ_i_^2^ weighted linear regression, as recommended for trace-level quantification.^53^

For assessment of the LOD, several common methods were applied, where the approaches of 3*S/m and 3*θ are more rigorous, incorporating blank sample signal variability rather than relying solely on a signal-to-noise (S/N) ratio threshold of >3.

Comparative analyses by^44^, who employed an analogous APPI-DMA-MS system, revealed notable discrepancies in LODs. Their study reported significantly lower LODs – using the 3*θ method – for TNT (0.05 pg in their work vs [0.21 pg in our work]) and DNT (0.01 pg [0.50 pg]), whereas the LOD for RDX was somehow comparable (1.4 pg [0.34 pg]). The improved RDX sensitivity in our study likely stems from the enhanced performance of the Sciex TQ6500+ mass spectrometer relative to the Qtrap 3200 used by McCulloch and colleagues. Critical differences in ionization chemistry might explain the divergent TNT and DNT results. McCulloch et al. analyzed the radical anions M^–•^ of DNT and TNT, which were not observable in our system due to the use of technical-grade nitrogen (99.5% purity) instead of ultrapure nitrogen (≥99.9992%). In our setup, ambient oxygen facilitated superoxide-mediated (O_2_^–^) proton abstraction, yielding the [M-H]^−^ ions for DNT and TNT.^1^ Ultrapure nitrogen not only enhances M^–•^ formation but also improves the separation performance of the DMA by minimizing vapor-induced ion clustering.^43^ Furthermore, it has to be acknowledged that McCulloch et al. analyzed the 2,6-DNT isomer instead of the 2,4-DNT isomer.

In a study of^51^, direct ionization of explosives by ESI or APPI (DESI/DAPPI) was compared. In contrast to our results for TNT (ESI is far more sensitive than APPI), they reported superior sensitivity of DAPPI over DESI (about 10-fold higher), while both ionization procedures performed comparably for RDX and PETN. While ionization mechanisms were consistent between studies, differences in ion source geometry likely contributed to the observed variations in analytical performance.^54^ Furthermore, Kauppila et al. applied different additives to the dopant in APPI (e.g., nitric acid, chloroform) and ESI (NaCl, HCl, TFA), observing increased intensities for RDX and PETN when adding nitric acid to the dopant for APPI.^51^

### Factors Influencing Detection of DNT

Our experiments revealed a significant discrepancy in DNT detection between liquid-phase analyses (detection was achieved) and filter-based measurements (no detection was observed) when using the ESI source. One plausible explanation lies in the transition from conventional electrospray ionization (ESI) for liquid samples to secondary electrospray ionization (SESI) for filter-based analysis. In SESI, vapor-phase analytes (e.g., DNT vapors desorbed from filters) are ionized in the gas-phase through interactions with ESI-generated ions or droplets. The process is not fully clarified and is also dependent on ion source design and properties of the analytes.^41^ This altered ionization process may have led to the formation of a different precursor ion (or multiple precursor ions) for DNT, compared to the observed precursor during liquid-phase ESI, rendering the originally optimized MRM transitions (based on the [M–H]^−^ ion) ineffective for detection. Furthermore, is has been reported that ions generated via ESI or SESI can exhibit different mobility spectra.^55^ Given that the DMA voltage was fixed for each analyte during the filter measurements, any substantial shift in DNT ion mobility could have caused ions to fall outside the selected mobility window, resulting in the detection deficiency of DNT in the filter samples.

Another difference between liquid-phase and filter-based analysis is the temperature: Thermal desorption from filters occurred at 200 °C, with the transferline maintained at 210 °C, whereas liquid-phase analysis involved a maximum temperature of only 160 °C. However, thermal degradation of DNT at 200 °C is not expected based on its known stability.^56^

While transferline efficiency is unlikely to be the main cause, since it is independent of the ion source, the configuration of the transferline connection differs significantly between ESI and APPI (see Figure 1A,B). In ESI, vapor-phase analytes enter the DMA orthogonally, whereas in APPI, they enter in-line with the DMA. These structural differences may influence gas flow dynamics and contribute to observed differences in detection performance. Further experiments are required to clarify the factors responsible for the challenges in detecting DNT from filters when using the ESI source.

### Soil Extracts

After evaluating the performance and limitations of both set-ups using laboratory explosive standards, the study was extended to the analysis of contaminated soils. For this purpose, standardized soil was fortified with explosives, extracted and analyzed using filter-based measurements. The soil extraction procedure applied in this study was previously optimized in our laboratory and tested on three different soil types (sandy loam, loamy sand and clay loam, see also Supporting Information). Analyses by LC-MS/MS confirmed the protocol’s robustness showing mean recoveries of 100 ± 6.8% (TNT) and 102 ± 5.1% (DNT) (Figure S2). Consequently, extraction inefficiency is excluded as a primary contributor to the observed recovery variations. For ESI-MS, soil extract concentrations exceeded expected values (mean ± SD: TNT 128 ± 9.8%, RDX 106 ± 9.9%), whereas APPI-MS analysis yielded lower concentrations than expected (mean ± SD: TNT 81.3 ± 11%, RDX 85.5 ± 15%). DNT exhibited the greatest variability (range: 37–112%, mean ± SD: 72.5 ± 23%), DNT analysis demonstrated the poorest sensitivity and reproducibility among all analytes. Both ionization methods show distinct analytical uncertainties, as reflected in the SD values. The reproducibility of the determinations performed with the ESI source was considered reliable, as standard deviations remained below 10%. In contrast, SD values for the samples analyzed by APPI are consistently higher implying greater variability in ionization efficiency and signal stability. A comparable trend was observed in liquid sample analyses, with replicate APPI measurements exhibiting greater variability, evidenced by higher standard deviations, compared to those obtained using ESI.

The divergent recoveries in the soil extracts might be attributed to ion source-specific matrix effects (enhancing effects for ESI, suppressing effects for APPI). Given the different ionization chemistries for ESI and APPI, this finding is not unexpected. The soil extraction procedure applied primarily recovered unbound organic compounds including fatty acids, hydroxyacids, alcohols, and unsaturated hydrocarbons.^57^ Fatty acids and phenolic compounds might interfere with the explosives by competing for superoxide ions, thereby decreasing the ionization efficiency of dopant-assisted APPI.^34^ In general, APPI is deemed to be less affected by matrix-induced ion suppression,^58^ however, this cannot be supported for our case and by our findings. The enhancing matrix effects we observed for the ESI source might be attributed to coextracted organic acids that can improve the formation of [M-H]^−^ -ions, leading to an increased ESI efficiency for soil extract samples.^59^ Laaniste et al.^54^ found an overall better sensitivity for ESI compared to APPI when analyzing various pesticides via LC/MS. In contrast, we observed analyte-dependent performance of both ion sources and a superior sensitivity of APPI toward DNT and 2-ADNT. However, both ionization methods are highly suitable for the detection of explosive trace amounts in contaminated soils with the ESI source exhibiting a slight advantage regarding sensitivity for TNT and RDX.

While absolute values should be interpreted with caution and would benefit from a more comprehensive calibration approach (e.g., matrix-matched calibration standards and/or stable isotope labeled internal standards), the system enables reliable and rapid qualitative assessment of soil contamination. The analyzed samples contained 1.2 μg/kg of TNT, 2.0 μg/kg RDX and 4 μg/kg of DNT – each yielding clear and robust signals, demonstrating the systems high sensitivity.

The direct extrapolation of these results to field-collected samples should be approached with caution. At ancient contaminated sites, explosives are frequently adsorbed to the soil matrix, which may limit the effectiveness of the extraction procedure. Although our experiments were conducted using standardized clay loam soil, real-world samples may exhibit markedly different matrix effects and extraction efficiencies depending e.g., on soil organic matter content.^7^ Nonetheless, the high sensitivity of the analytical system allows for a reliable qualitative assessment of contamination, even when only a fraction of the explosives is extracted. When combined with a sampling strategy that accounts for the inherent spatial heterogeneity of explosive residues in soils, this approach holds strong potential for the detection of trace explosive compounds associated with unexploded ordnance (UXOs), where contaminants may be present at ultratrace levels in the μg/kg range.^60^

In the context of environmental monitoring, where method detection limits are typically in the mg/kg range,^61^ the system still offers a significant advantage: the fast analysis time of less than 3 min. The primary factor limiting throughput remains the analyte extraction step, which could be further optimized to accelerate the overall process. Future work should focus on streamlining extraction protocols to enhance the system’s speed and suitability for high-throughput screening.

For a final overview, Table 8 provides a concise summary of the analytical performance of the two ion sources when analyzing explosives from liquid or filter samples, including the analysis of soil extracts.

**Table 8:** Overview on Analytical Performance of ESI and APPI for the Different Analytes and Application Scenarios^a^

Analyte	Sensitivity				Analytical Uncertainty (SD)	
	Liquid		Filters		Filters (Soil Samples)	
	ESI	APPI	ESI	APPI	ESI	APPI
TNT	**	*	***	**	9.8	11
RDX	***	**	**	*	9.9	15
2.4-DNT	*	***	-	*	-	23
2A-4.6-DNT	*	***	--	--	--	--
PETN	***	*	--	--	--	--

a Sensitivity: * low, ** medium, *** high; - no signal; -- not tested.

## Conclusion

This study demonstrates that the tested DMA-MS-system is well-suited for the reliable and rapid trace detection of explosives in soil samples. The choice of ionization source – ESI versus APPI – was found to critically influence system performance, with distinct differences in sensitivity, selectivity, and susceptibility to matrix effects. All of these factors should already be taken into account during method development. ESI provided superior sensitivity for PETN, RDX and TNT but failed to detect DNT via thermal desorption, possibly due to limitations in ionization efficiency. In contrast, APPI showed enhanced sensitivity toward DNT and 2A-DNT in liquid samples and enabled the detection of DNT from filters, though it suffered from pronounced signal suppression in complex soil matrices. Nevertheless, both ionization principles enabled fast and sensitive detection of explosive residues in soils in the μg/kg range. Depending on the application scenario, the optimal ionization strategy has to be chosen in order to facilitate the detection of the desired analytes with the necessary sensitivity. Future investigations will focus on the filter-based detection of further explosive analytes such as PETN, HMX or 2A-DNT - as a marker for TNT contamination in soils - further expanding the systems utility for environmental screening and landmine detection tasks.

## Supplementary Material



## Notes

Signals for different amounts of DNT (filter measurements ESI) (Figure S1); relative standard deviations for each calibration level (Table S1); description: verification of the performance of the extraction procedure; mass transitions and MS settings for TNT and DNT for LC-MS/MS (Table S2); boxplots of recoveries of DNT and TNT in soil extracts (Figure S2) (PDF)

## §

School of Mechanical Engineering and Safety Engineering, University of Wuppertal, Gaußstraße 20, 42119 Wuppertal, Germany
