# Supplementary material for: Comparison of ESI and APPI for the Detection of Nitro Explosives Using Differential Mobility Mass Spectrometry
Source: ACS Omega. 2026 Aug 14;11(33):50471–82. doi: 10.1021/acsomega.6c06952 (PMC13520020; doi:10.1021/acsomega.6c06952)
Supplement: Supplementary file 1 [file ao6c06952_si_001.pdf]

Supporting Information

Comparison of ESI and APPI for the Detection of Nitro Explosives using Differential Mobility Mass Spectrometry

Vanessa Lünsmann[*,†], René Breuch[†,‡], Silvia Ann Marit Herrmann[‡,§], Kostyantin Konstantynovski [†]

[†]*Institute for the Protection of Terrestrial Infrastructures, German Aerospace Center, Rathausallee 12, 53757 Sankt Augustin, Germany*

[‡]*Hochschule Bonn-Rhein-Sieg, University of Applied Sciences, Institute of Safety and Security Research, von-Liebig-Str. 20, 53359, Rheinbach, Germany*

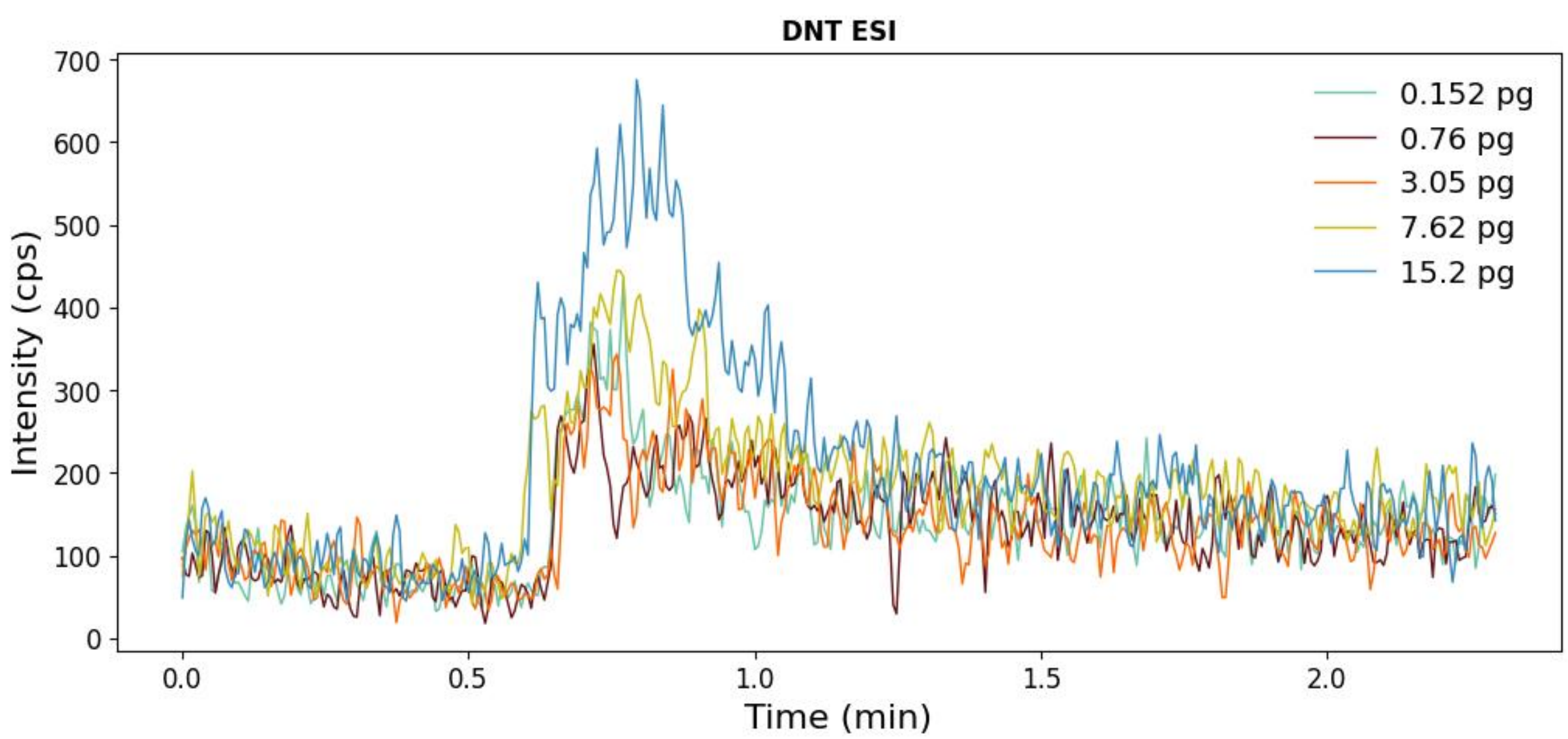

Figure S 1: Signals for different amounts of DNT analysed via ESI by thermo desorption from filters

Table S 1: Relative standard deviations for each calibration level

Analyte	Standard [pg]	ESI<br>RSD [%]	APPI<br>RSD [%]
TNT	0.0993	12.3	-
	0.248	27.4	-
	0.497	23.1	44.1
	0.993	34.2	27.9
	1.99	29.5	17.6
	3.48	22.1	15.3
	4.97	9.2	14.5
	7.45	15.9	12.1
	9.93	11.5	15.1
RDX	0.298	25.5	-
	0.744	18.2	-
	1.49	13.7	32.1
	2.98	15.2	26.6
	5.95	9.3	21.2
	10.4	20.3	24.3
	14.9	8.0	21.0
	22.3	10.2	11.6
	29.8	10.0	11.6
DNT	0.152	-	-
	0.381	-	-
	0.762	-	26.1
	1.52	-	28.5
	3.05	-	13.1
	5.33	-	22.3

	7.62	-	14.2
	11.4	-	25.8
	15.2	-	21.3

Verification of the performance of the extraction procedure

Spiking and extraction: Three different standardized soils (SP2.4N - soil type (USDA) clay loam, SP2.1 – loamy sand, SP2.2 – sandy loam) were obtained from LUFA Speyer (Speyer, Germany). The soils were fortified in triplicates with TNT and 2,4-DNT at each 0.040 mg/kg and 0.180 mg/kg. The fortification and extraction procedure are described in the materials and methods section of the publication.

Determination by LC-MS: Soil extracts were analysed using a HPLC Agilent 1260 Infinity (Agilent Technologies; column oven G131A TCC, autosampler G1330B FC/ALS Therm, G1329A ALS, degasser G1379B, binary pump G1312A) coupled to a QTRAP 5500 MS (Sciex) with an ESI-source. For analysis, an in-line guard column (Security Guard Cartridge, C_{18} 2 x 2.0 mm, Phenomenex) and the column Luna 3 µm C_{18}(2) 100 Å, 150mm x 2mm (Phenomenex) were used. Mobile phase A was 10 mM ammonium acetate (Carl Roth GmbH & Co. KG) in LC-MS grade water (Honeywell, Riedelde-Haen), LC-MS grade acetonitrile (CHEMSOLUTE) was mobile phase B. Flow rate: 0.3 mL/min, injection volume 10 µL, column oven temperature: 40 °C, autosampler temperature: 20 °C. The applied gradient started at 40% B increasing to 95% B within 6 min. 95% B was hold for 2.5 min and the system was left for re-equilibration at 40% B for 8 min. The turbo ion source of the mass spectrometer was operated in negative mode with -4500V, at 450 °C, 20 psi curtain gas and 60 psi for gas 1 and 2. Monitored mass transitions for TNT and DNT are given in Table S 2.

Table S 2: Mass transitions and MS settings for TNT and DNT (LC-MS/MS)

Analyte	Q1 (Da)	Q3 (Da)	Dwell time (ms)	DP (V)	EP (V)	CE (V)	CXP (V)
2.4.6-TNT	226	46*	100	-45	-8	-73	-8
$[M-H]^-$	226	196	100	-45	-8	-19	-8
2.4-DNT	181	46*	100	-45	-8	-52	-7
$[M-H]^-$	181	135	100	-45	-8	-26	-11

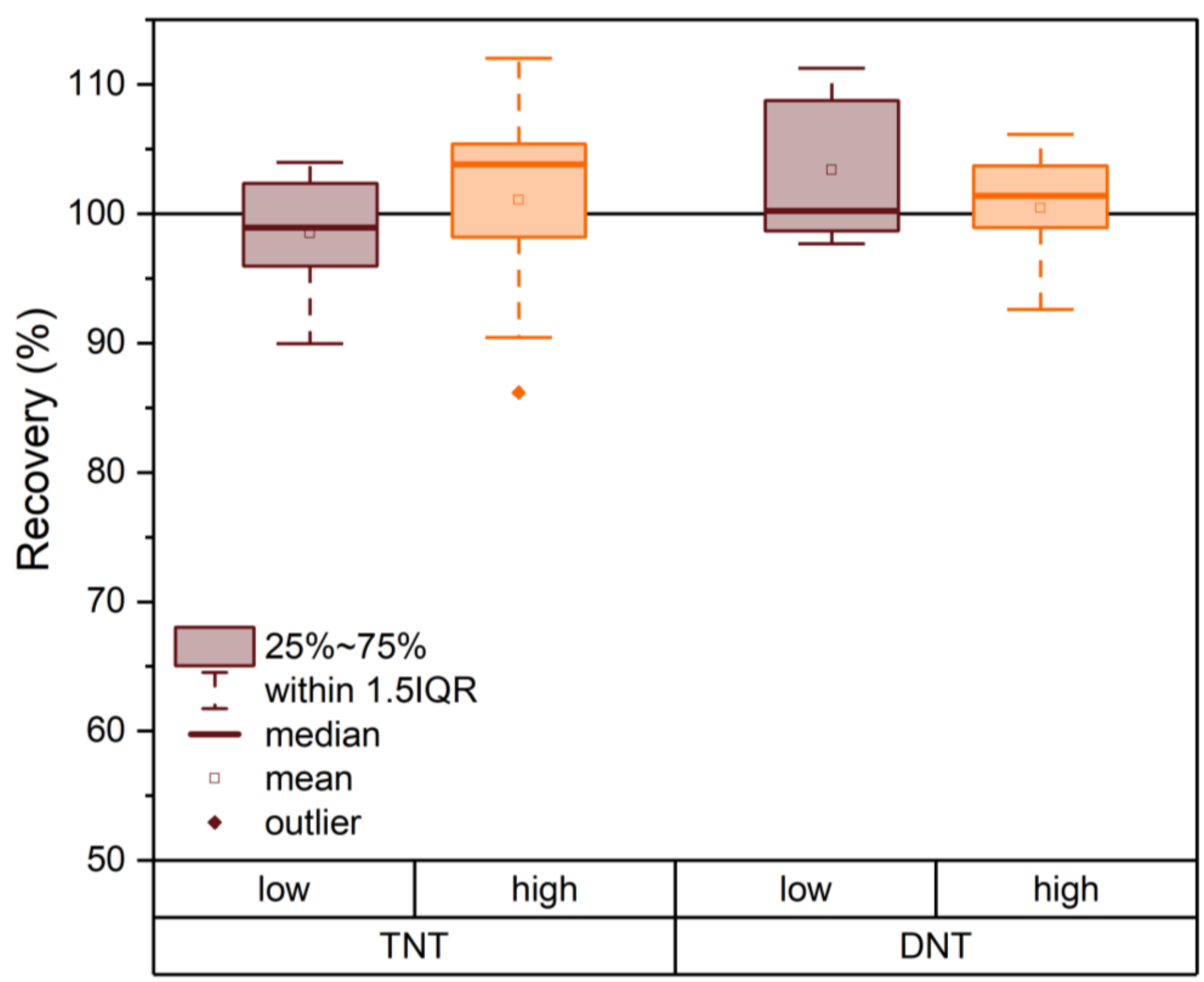

Figure S 2: Boxplots of recoveries of DNT and TNT in extracts of soils spiked with each 0.040 mg/kg (low) and 0.180 mg/kg (high). N=9 for each category.
